# Supplementary material for: Unraveling Depressive Symptomatology and Risk Factors in a Changing World
Source: Int J Environ Res Public Health. 2023 Aug 13;20(16):6575. doi: 10.3390/ijerph20166575 (PMC10454694; doi:10.3390/ijerph20166575)
Supplement: Supplementary file 1 [file ijerph-20-06575-s001.zip › ijerph-2415725-supplementary.pdf]

Table S1 - Univariate Multinomial Logistic Regression model for the association of sociodemographic, lifestyle and health characteristics and depression levels

		Normal vs Mild		
		**Relative Risk ratio**	**p-value**	**[95% CI]**
**Gender**				
	Male	Ref	-	-
	Female	3.09	<0.001	[2.22-4.30]
**Age-group**				
	25-34	Ref	-	-
	35-44	3.55	0.010	[1.36-9.27]
	45-54	2.86	0.029	[1.11-7.35]
	55-64	5.18	<0.001	[2.06-13.03]
	65-74	5.48	<0.001	[2.17-13.82]
	≥ 75 years	8.91	<0.001	[3.54-22.42]
**NUTSII**				
	LVT	Ref	-	-
	Norte	2.07	0.001	[1.36-3.15]
	Centro	2.45	<0.001	[1.58-3.80]
	Alentejo	1.88	0.056	[0.98-3.61]
	Algarve	2.91	0.005	[1.38-6.15]
	Azores	2.63	<0.001	[1.57-4.40]
	Madeira	1.98	0.020	[1.11-3.53]
**Marital Status**				
	Married	Ref	-	-
	Other	1.09	0.546	[0.83-1.42]
**Education level**				
	0-4 years	Ref	-	-
	5-9 years	0.50	<0.001	[0.36-0.71]
	10-12 years	0.37	<0.001	[0.25-0.53]
	>12 years	0.22	<0.001	[0.14-0.34]
**Employment status**				
	Employed full/part-time	Ref	-	-
	Retired	2.83	<0.001	[2.07-3.87]
	Other	4.88	<0.001	[3.43-6.95]
**Income perception**				
	Living comfortably in the present income	Ref	-	-
	Living in the present income	2.31	<0.001	[1.58-3.38]
	Finding difficult in the present income	4.85	<0.001	[3.16-7.44]
	Finding it very difficult in the present income	10.70	<0.001	[6.54-17.50]
**Household size**				
	1 person	Ref	-	-
	2 persons	0.63	0.007	[0.45-0.88]
	3 or more persons	0.44	<0.001	[0.31-0.61]
**BMI (kg/m²)**				
	Underweight/Normal	Ref	-	-
	Overweight	1.22	0.230	[0.88-1.69]
	Obese	1.94	<0.001	[1.38-2.74]
**Alcohol intake**				
	Never	Ref	-	-
	Daily	0.31	<0.001	[0.21-0.45]
	Occasional	0.37	<0.001	[0.27-0.51]
**Smoking Habits**				
	Never	Ref	-	-
	Past smoker	0.51	<0.001	[0.35-0.74]
	Current/Occasionally smoker	0.66	0.041	[0.45-0.98]
**Regular Exercise**				
	Never	Ref	-	-

	Yes	0.33	<0.001	[0.23-0.50]
	Ocasionally	0.44	<0.001	[0.31-0.62]
**Multimorbidity (self-reported)**				
	No	Ref	-	-
	Yes	3.59	<0.001	[2.67-4.82]
**COVID-19 infection**				
	No	Ref	-	-
	Yes	0.73	0.254	[0.43-1.25]
			**Normal vs Moderate/Severe**	
		**Relative Risk ratio**	**p value**	**[95% CI]**
**Gender**				
	Male	Ref	-	-
	Female	3.28	<0.001	[2.18-4.92]
**Age-group**				
	25-34	Ref	-	-
	35-44	1.18	0.760	[0.40-3.50]
	45-54	1.39	0.518	[0.51-3.78]
	55-64	5.43	<0.001	[2.16-13.67]
	65-74	3.36	0.012	[1.30-8.68]
	≥ 75 anos	5.57	<0.001	[2.17-14.29]
**NUTSII**				
	LVT	Ref	-	-
	Norte	1.30	0.232	[0.84-2.00]
	Centro	1.21	0.441	[0.75-1.96]
	Alentejo	1.35	0.396	[0.67-2.72]
	Algarve	0.27	0.195	[0.04-1.97]
	Azores	1.55	0.129	[0.88-2.74]
	Madeira	1.18	0.623	[0.61-2.26]
**Marital Status**				
	Married	Ref	-	-
	Other	1.01	0.971	[0.73-1.39]
**Education level**				
	0-4 years	Ref	-	-
	5-9 years	0.55	0.003	[0.37-0.82]
	10-12 years	0.35	<0.001	[0.22-0.54]
	>12 years	0.07	<0.001	[0.03-0.16]
**Employment status**				
	Employed full/part-time	Ref	-	-
	Retired	5.12	<0.001	[3.29-7.98]
	Other	9.73	<0.001	[6.07-15.60]
**Income perception**				
	Living comfortably in the present income	Ref	-	-
	Living in the present income	2.81	<0.001	[1.63-4.82]
	Finding difficult in the present income	8.94	<0.001	[5.09-15.70]
	Finding it very difficult in the present income	19.70	<0.001	[10.62-36.56]
**Household size**				
	1 person	Ref	-	-
	2 persons	0.59	0.007	[0.40-0.87]
	3 or more persons	0.31	<0.001	[0.21-0.47]
**BMI (kg/m²)**				
	Underweight/Normal	Ref	-	-
	Overweight	1.15	0.478	[0.79-1.67]
	Obese	1.58	0.031	[1.04-2.38]
**Alcohol intake**				
	Never	Ref	-	-
	Daily	0.26	<0.001	[0.16-0.42]
	Occasional	0.30	<0.001	[0.20-0.45]
**Smoking Habits**				

	Never	Ref	-	-
	Past smoker	0.52	0.004	[0.33-0.81]
	Current/Occasionally smoker	0.68	0.041	[0.43-1.10]
**Regular Exercise**				
	Never	Ref	-	-
	Yes	0.40	<0.001	[0.26-0.63]
	Ocasionally	0.42	<0.001	[0.27-0.65]
**Multimorbidity (self-reported)**				
	No	Ref	-	-
	Yes	3.58	<0.001	[2.51-5.11]
**COVID-19 infection**				
	No	Ref	-	-
	Yes	0.95	0.875	[0.53-1.71]
